# The dual specificity phosphatase 2 gene is hypermethylated in human cancer and regulated by epigenetic mechanisms

**DOI:** 10.1186/s12885-016-2087-6

**Published:** 2016-02-01

**Authors:** Tanja Haag, Antje M. Richter, Martin B. Schneider, Adriana P. Jiménez, Reinhard H. Dammann

**Affiliations:** Institute for Genetics, Justus-Liebig-University, Heinrich-Buff-Ring 58-62, D-35392 Giessen, Germany; Universities of Giessen and Marburg Lung Center, 35392 Giessen, Germany

**Keywords:** Cancer, Dual specificity phosphatase 2, Epigenetic, Merkel cell carcinoma, CTCF, DNA methylation

## Abstract

**Background:**

Dual specificity phosphatases are a class of tumor-associated proteins involved in the negative regulation of the MAP kinase pathway. Downregulation of the *dual specificity phosphatase 2* (*DUSP2*) has been reported in cancer. Epigenetic silencing of tumor suppressor genes by abnormal promoter methylation is a frequent mechanism in oncogenesis. It has been shown that the epigenetic factor CTCF is involved in the regulation of tumor suppressor genes.

**Methods:**

We analyzed the promoter hypermethylation of *DUSP2* in human cancer, including primary Merkel cell carcinoma by bisulfite restriction analysis and pyrosequencing. Moreover we analyzed the impact of a DNA methyltransferase inhibitor (5-Aza-dC) and CTCF on the epigenetic regulation of *DUSP2* by qRT-PCR, promoter assay, chromatin immuno-precipitation and methylation analysis.

**Results:**

Here we report a significant tumor-specific hypermethylation of *DUSP2* in primary Merkel cell carcinoma (*p* = 0.05). An increase in methylation of *DUSP2* was also found in 17 out of 24 (71 %) cancer cell lines, including skin and lung cancer. Treatment of cancer cells with 5-Aza-dC induced *DUSP2* expression by its promoter demethylation, Additionally we observed that CTCF induces *DUSP2* expression in cell lines that exhibit silencing of *DUSP2*. This reactivation was accompanied by increased CTCF binding and demethylation of the *DUSP2* promoter.

**Conclusions:**

Our data show that aberrant epigenetic inactivation of *DUSP2* occurs in carcinogenesis and that CTCF is involved in the epigenetic regulation of *DUSP2* expression.

**Electronic supplementary material:**

The online version of this article (doi:10.1186/s12885-016-2087-6) contains supplementary material, which is available to authorized users.

## Background

Dual specificity phosphatases (DUSPs) are negative regulators of mitogen-activated protein kinases (MAPK) that regulate proliferative signaling pathways, which are often activated in cancer [1–3]. DUSP2 encodes a dual-specificity phosphatase that inactivates ERK1/2 and p38 MAPK [4, 5]. DUSP2 has also been found to regulate p53- and E2F1-regulated apoptosis [6, 7]. Previously it has been reported that *DUSP2* expression is markedly reduced or completely absent in many human cancers [8, 9].

Epigenetic silencing of tumor suppressor genes (TSG) is one of the most relevant molecular alteration that occurs during carcinogenesis [10]. Promoter hypermethylation of TSG occurs in cancer through methylation at the DNA level at C5 of cytosine (5mC), when found as a dinucleotides with guanine. DNA methylation in CpG islands of TSG leads to epigenetic silencing of the according transcript [11, 12]. The ten-eleven-translocation methylcytosine dioxygenases (TET1-3) catalyze the oxidation of 5mC and generate cytosine derivatives including 5-hydroxymethylcytosine (5hmC) [13, 14]. TET proteins are involved in diverse biological processes, as the zygotic epigenetic reprogramming, hematopoiesis and the development of leukemia [15–17]. The frequency of 5hmC suggests that these modified cytosine bases play an important role in epigenetic gene regulation [18]. Aberrant levels of 5hmC have been reported in human cancer [19, 20]. Recently it has been shown that TET proteins bind the CCCTC binding factor (CTCF) [21]. CTCF is associated with altered expression of tumor suppressor genes, such as E-cadherin (CDH1), retinoblastoma 1, RASSF1A, CDKN2A/p16 and TP53 [22–25]. It has also been postulated, that CTCF itself acts as a tumor suppressor [26, 27].

Here we analyzed the epigenetic inactivation and regulation of the *dual specificity phosphate 2* (DUSP2) in human cancers. Our data show that *DUSP2* is aberrantly methylated in primary Merkel cell cancer and in different human cancer cell lines. Moreover, we observed that 5-Aza-dC and CTCF induce *DUSP2* expression by its promoter demethylation.

## Methods

### Primary tissues and cell lines

The analyzed primary tissues include 22 Merkel cell carcinoma [28, 29], 20 pheochromocytoma [30, 31], six small cell lung cancer [32], 12 breast carcinoma [25, 33] and 12 benign nevus cell nevi [34]. RNA samples from normal tissues (liver, breast, kidney and lung) were obtained from Agilent Technologies (Santa Clara, USA). All patients signed informed consent at initial clinical investigation. The study was approved by local ethic committees (City of Hope Medical Center, Duarte, USA or Martin-Luther University, Halle, Germany). All cell lines were cultured in a humidified atmosphere (37 °C) with 5 % CO_2_ and 1× Penicillin/Streptomycin in according medium. Cells were transfected with 4 μg of constructs on 3.5 cm plates, using Polyethylenimine or X-tremeGENE HP (Roche Applied Science, Germany). TREx293 cells, that stably express the Tet repressor (LifeTechnologies), were transfected with the expression vector pcDNA4TO-CTCF and selected with Zeocin™ (Invitrogen). CTCF was induced by tetracycline (5 μl/ml of a 1 mg/ml stock) over 48 h.

### Methylation analysis

DNA was isolated by phenol-chloroform extraction and then bisulfite treated prior to combined bisulfite restriction analysis (COBRA) and pyrosequencing [35]. 200 ng were subsequently used for semi nested PCR with primer DUSP2BSU2 (GGGATTTGTATTTGAGAAGTTGGGTTTT) and DUSP2BSL2 (CCTCCAACCCCATAACCACC) in a first PCR. For the second PCR DUSP2BSU1 (GTTTTTTTTYGGTGTGTTGGTTTT) and the 5′-biotinylated primer DUSP2BSBIO (CCTCCAACCCCATAACCACC) were used. Products were digested with 0.5 μl *Taq*I (Fermentas) 1 h at 65 °C and resolved on 2 % TBE agarose gel. Methylation status was quantified utilizing the primer DUSP2BSSeq1 (TTTTGTTTTTTTTTTTAATTTTTTTT) and DUSP2BSSeq2 (GTTTTTTTGTTTTGTTTTTGTATGGTGTT) and PyroMark Q24 (Qiagen). Five CpGs are included in the analyzed region with primer DUSP2BSSeq1 and seven in the region analyzed with primer DUSP2BSSeq2. For in vitro methylation of genomic DNA we used M.SssI methylase (NEB).

### Expression analysis

RNA was isolated using the Isol-RNA lysis procedure (5′Prime). 25 μg of breast, kidney, liver and lung RNA of normal human samples were obtained from Agilent Technologies (Santa Clara, CA, USA). RNA was DNase (Fermentas GmbH, St.Leon-Rot, Germany) digested and then reversely transcribed [36]. RT-PCR was performed with primers listed in Additional file 1: Table S1. Quantitative PCR (qRT-PCR) was performed in triplicates with SYBR® Select Master Mix (Life Technologies) using Rotor-Gene 3000 (Corbett Research, Qiagen).

### Promoter assay

The *DUSP2* promoter was amplified with primers DUSP2BglIIU1: CAGATCTGAGTGGCTTGGGACAGGTCA and DUSP2PromL1: CAGCAGCAGCGTGCGTTCCG from genomic DNA. The 454 bp promoter fragment was cloned into the *Bgl*II sites of pRLnull (Promega, Mannheim, Germany) and sequenced. In vitro methylation of the promoter construct was done with M.SssI methylase (NEB, Frankfurt, Germany). HEK293 were transfected with 1 μg of pRL-DUSP2 promoter construct and 0.35 μg of pGL3 control vector (Promega, Mannheim, Germany). Cells were isolated 24 h after transfection and studied using Dual-Luciferase Reporter Assay (Promega, Mannheim, Germany).

### Depletion of CTCF by RNAi

Five small interfering RNAs against CTCF: HSS173820 (Stealth siRNA), HSS116456 (Stealth siRNA), HSS116455 (Stealth siRNA), siCTCF1: UCACCCUCCUGAGGAAUCACCUUAA, siCTCF2: GAUGCGCUCUAAGAAAGAA and a control siRNA: CUACGAUGAAGCACUAUUATT were obtained from Invitrogen (Carlsbad, CA, USA) and have been characterized previously [37, 38]. Cells were transfected with a pool of five specific siRNAs against CTCF or a control siRNA according to the manufacturer manual using Lipofectamin RNAiMax from Invitrogen (Carlsbad, CA, USA) on two consecutive days and incubated for a total of 96 h. RNA and protein was isolated.

### Chromatin immunoprecipitation (ChIP)

Cells were fixed using 37 % formaldehyde (CalBiochem) with a final concentration of 1 % for 10 min at room temperature. Incubation of 1/7 volume of 1 M glycine for 5 min stopped the fixation process. Cells were washed with PBS and harvested in PBS + 1 mM PMSF. After centrifugation for 2 min at 2000 rpm at 4 °C the supernatant was removed and cells were lysed using 1 ml SDS lysis buffer (0,5 % SDS, 10 mM EDTA, 50 mM Tris HCl pH 8.1) supplemented with protease inhibitors (Complete Mini, Roche) per 10^7^ cells for 10 min on ice. After sonification (400-800 bp) the samples were centrifuged for 10 min at 4 °C and maximum speed. The supernatant was diluted 1:10 with dilution buffer (0,01 % SDS, 1,1 % Triton X-100, 1.2 mM EDTA, 16.7 mM Tris/HCl pH 8.1, 167 mM NaCl) and 1 ml of the dilution was used for each ChIP. 10 % of the chromatin used for one ChIP was preserved as an input sample and stored at -20 °C. The lysate was pre-cleared by rotation at 4 °C for 2 h using 1 ml of the dilution and 20 μl ProteinG Plus/ProteinA Agarose (Calbiochem). After centrifugation at 4 °C for 1 min at 2000 rpm the supernatant was incubated with the corresponding antibody: IgG (46540; Santa Cruz Biotechnologie), Histon H3 (1791; Abcam), α-CTCF-(N2.2, [39]) rotating overnight at 4 °C. Binding of the immune-complexes occurs afterwards by incubation of the chromatin with 20 μl of ProteinG Plus/Protein A agarose for 2 h at 4 °C. After incubation the beads were washed for 5 min rotating at 4 °C one time with low salt buffer (0,05 % SDS, 1 % TritonX100, 2 mM EDTA, 20 mM Tris/HCl pH 8.1, 150 mM NaCl), one time with high salt buffer (0,05 % SDS, 1 % TritonX100, 2 mM EDTA, 20 mM Tris/HCl pH 8.1, 500 mM NaCl), one time with LiCl buffer (0,25 M LiCl, 1 % NP40, 1 % Deoxycholat, 1 mM EDTA, 10 mM Tris/HCl pH 8.1) and two times with TE buffer (10 mM Tris, 1 mM EDTA pH 8.0). Chromatin bound to beads and input material were resuspended in 100 μl TE buffer, 1 μl of 10 mg/ml RNase was added followed by an incubation of 30 min at 37 °C. 5 μl of 10 % SDS, 1 μl of 20 mg/ml Proteinase K was added and incubated for further 2-4 h at 37 °C. The reverse crosslink was performed by incubating the samples over night at 65 °C. DNA was recovered by using QIAquick PCR Purification Kit (Qiagen) and PCR amplification with the following primer: DUSP2CIPU1: TTTGAGGGCCTTTTCCGCTACAAGAG, DUSP2CIPL1: GCCTCCGCTGTTCTTCACCCAGTC, DUSP2CIPU2: GGGTGGGCGCAAAAACGGAGGG, DUSP2CIPL2: CCGGGGCACCATACAAGGGCAGA, DUSP2CIPU3: GGCCACGTCAC CCTCTCAGTGTCTC, DUSP2CIPL3: GCCTCAGCCAAGTTGCCCAGACA. PCR was done with U1/L1 (233 bp), U2/L2 (236 bp) and U3/L3 (120 bp) for positive site, DUSP2 promoter site and negative site, respectively. Quantitative PCR was performed in triplicates with SYBR® Select Master Mix (Life Technologies) using Rotor-Gene 3000 (Corbett Research, Qiagen).

### Methylated DNA-immunoprecipitation (MeDIP)

MeDIP was performed according to the protocol of Mohn et al. [40] with antibodies: IgG (46540; Santa Cruz Biotechnologie), 5mC (MAb-081-010; Diagenode) and 5hmC (MAb-31HMC-020; Diagenode). The following primers were used for semi quantitative PCR amplification: DUSP2CIPU2: GGGTGGGCGCAAAAACGGAGGG, DUSP2CIPL2: CCGGGGCACCATACAAGGGCAGA, bACTRTFW: CCTTCCTTCCTGGGCATGGAGTC, bACTRTFW: CGGAGTACTTGCGCTCAGGAGGA.

### Western blot

Cell lysates were resolved in SDS-PAGE, immunoblotted, and probed with primary anti-CTCF (N2.2) and anti-GAPDH antibody (GAPDH rabbit polyclonal IgG FL-335, Santa Cruz) [39]. Afterwards an incubation with HRP-coupled secondary antibodies followed. Immunocomplexes were detected by enhanced chemiluminescence reagent (Western Chemiluminescent Immobilon HRP-Substrate, Millipore) according to the manufacturer’s instructions.

### Constructs

CTCF and BORIS were generous gifts from Rainer Renkawitz (Justus-Liebig-University, Giessen, Germany) and deletions and mutations were generated with QuikChange Lightning Site-Directed Mutagenesis Kit (Promega, Heidelberg, Germany) with the primers listed in Additional file 2: Table S2.

### Statistical evaluation

Statistical analysis was performed using Excel (Microsoft, Redmond, USA) and GraphPad Quick Calcs (GraphPad Software, La Jolla, USA). Data are represented as mean ± standard deviation. Unpaired t-test was used to determine significant differences between groups. For statistical analysis of methylation differences between Merkel cell carcinoma and benign controls a two tailed Fisher exact test was performed. All reported *p*-values are considered significant for p ≤ 0.05.

## Results

### Aberrant promoter methylation of *DUSP2* in human cancer

**Fig. 1:**
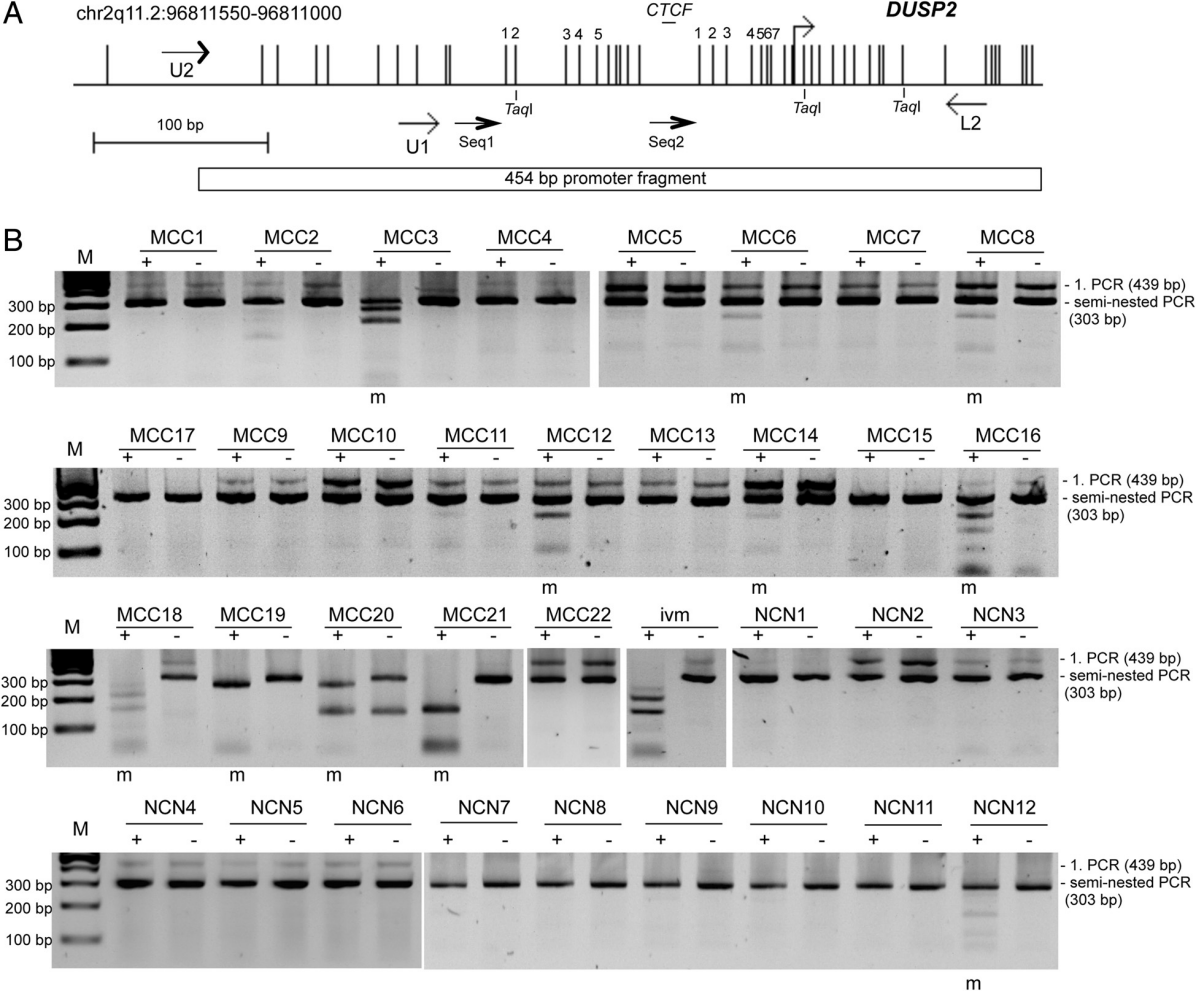
Hypermethylation of *DUSP2* in primary Merkel cell carcinoma (MCC). **a**. Structure of the *DUSP2* CpG island promoter on chromosome 2q11.2. Vertical lines indicate CpGs and the transcriptional start site is marked. A CTCF motif sequence (GGCAGAGCA; CTCFBSDB2.0) is marked [47]. Primers used for COBRA and sequencing (Seq1 and Seq2) are depicted by arrows. *TaqI* restriction sites for COBRA and CpGs analyzed by pyrosequencing are indicated. The 454 bp *DUSP2* fragment for the luciferase promoter assay is indicated. **b**. Methylation of DUSP2 in MCC (m = methylated). For COBRA bisulfite-treated DNA from MCC, benign nevus cell nevi (NCN) and in vitro methylated DNA (*ivm*) was amplified by semi-nested PCR. First and second PCR products are indicated (439 bp and 303 bp, respectively). Products were digested with *Taq*I (+) or mock digested (-) and resolved on 2 % agarose gels with a 100 bp marker (M)

Previously it has been shown that expression of the MAPK-specific phosphatase DUSP2 is markedly reduced or completely absent in many human cancers and that its level of expression inversely correlates with cancer malignancy [8]. Here we aimed to dissect the epigenetic mechanisms involved in the aberrant downregulation of *DUSP2* in carcinogenesis. *DUSP2* is located on 2q11.2 (Fig. 1a) and contains a 1011 bp CpG island in its promoter region (chr2: 96810444-96811454, UCSC genome browser). We analyzed the promoter hypermethylation of *DUSP2* in primary tumors including Merkel cell carcinoma (MCC), pheochromocytoma, small cell lung cancer and breast carcinoma by COBRA (Fig. 1 and Additional file 3: Figure S1). In 12 breast carcinoma, 20 pheochromocytoma and six small cell lung cancer samples the *DUSP2* promoter was unmethylated (Additional file 3: Figure S1). Interestingly, 10 out of 22 (45 %) Merkel cell carcinoma showed a *DUSP2* hypermethylation (Fig. 1b). MCC is a rare but aggressive cutaneous malignancy. In the control tissue (benign nevus cell nevi) only one out of 12 (8 %) analyzed samples exhibited a *DUSP2* hypermethylation (NCN12; Fig. 1b). Thus in MCC a significant tumor-specific hypermethylation of *DUSP2* was detected (*p* = 0.05, two tailed Fisher exact test).

**Fig. 2:**
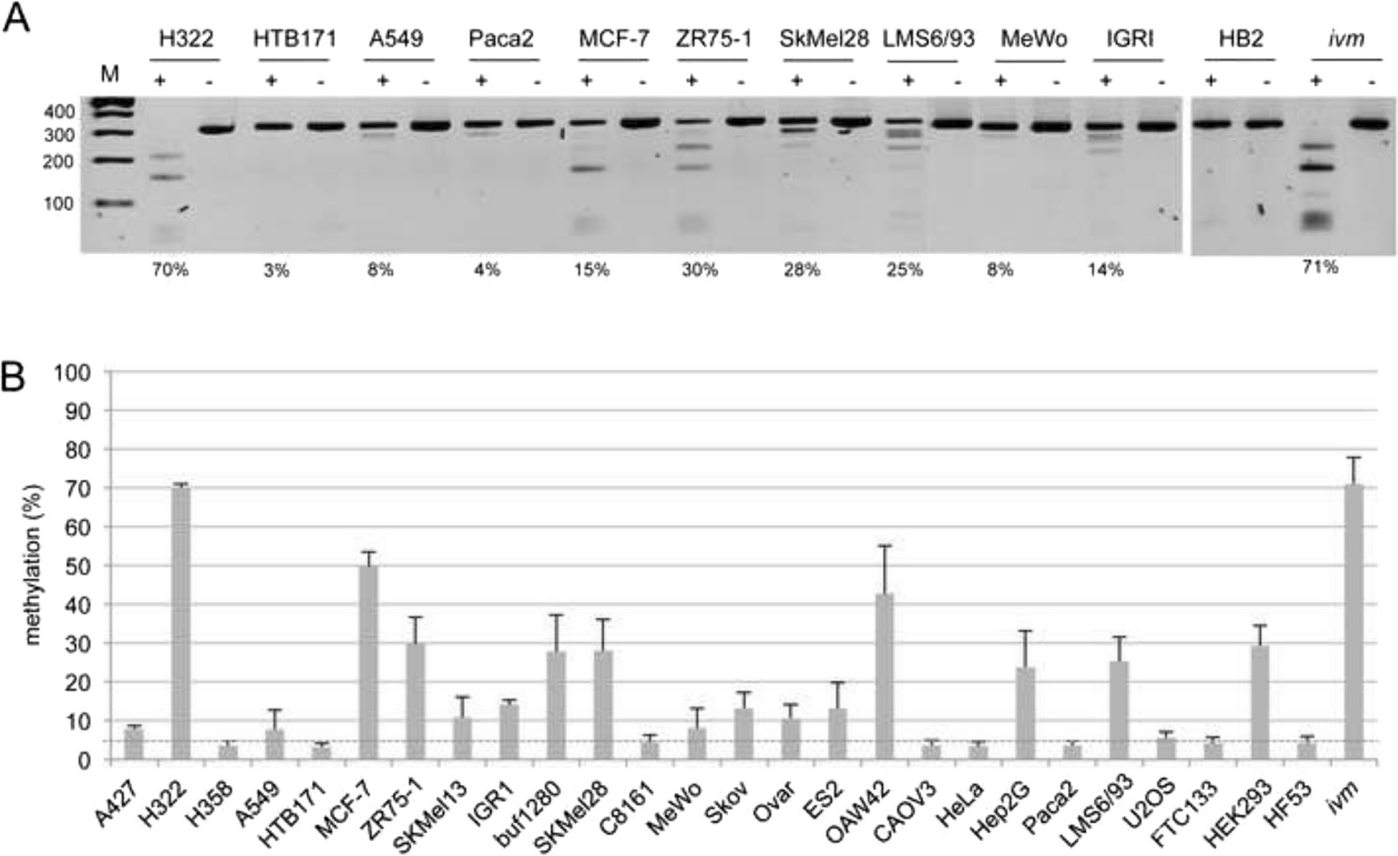
Promoter hypermethylation of *DUSP2* in human cancers. **a**. Combined bisulfite restriction analysis (COBRA) of *DUSP2*. Bisulfite-treated DNA from the indicated cancer cell lines, normal epithelial breast cells (HB2) and in vitro methylated DNA (*ivm*) was amplified, digested with *Taq*I (+) or mock digested (-) and resolved on 2 % agarose gels with a 100 bp marker (M). Methylation levels obtained from pyrosequencing are indicated in percentage. **b**. Bisulfite pyrosequence analysis of *DUSP2* in human cells. The mean methylation levels of five CpGs were analyzed by pyrosequencing (Seq1). The analysis included the results of three independent experiments. The dashed line marks 5 % threshold

To reveal the epigenetic status of *DUSP2* in human cancers in more detail, we have analyzed the methylation of its promoter in several human cancer cell lines by COBRA (Fig. 2a) and by pyrosequencing (Fig. 2b). Human fibroblasts (HF53) and the HB2 mammary luminal epithelial cells are unmethylated (methylation level <5 %) (Fig. 2a and b). We detected increased *DUSP2* methylation (level ≥5 %) in lung cancer (A427, H322, A549), breast cancer (MCF-7, ZR75-1), melanoma (SKMel13, IGR1, buf1280, SKMel28, MeWo), ovarian cancer (Skov, Ovar, ES2, OAW42), hepatocarcinoma (Hep2G) and sarcoma (LMS6/93, U2OS) cell lines (Fig. 2a and b). Lung cancer cell lines H358 and HTB171, melanoma C8161, ovarian cancer CAOV3, pancreatic cancer PaCa2, thyroid cancer FTC133 and HeLa were rather unmethylated (<5 %). Furthermore the embryonic kidney cell line HEK293 showed an aberrant *DUSP2* promoter methylation (29 %). In summary, 17 out of 24 (71 %) human cancer cell lines exhibited increased *DUSP2* promoter methylation.

### Decreased expression of *DUSP2* is associated with its promoter hypermethylation

**Fig. 3:**
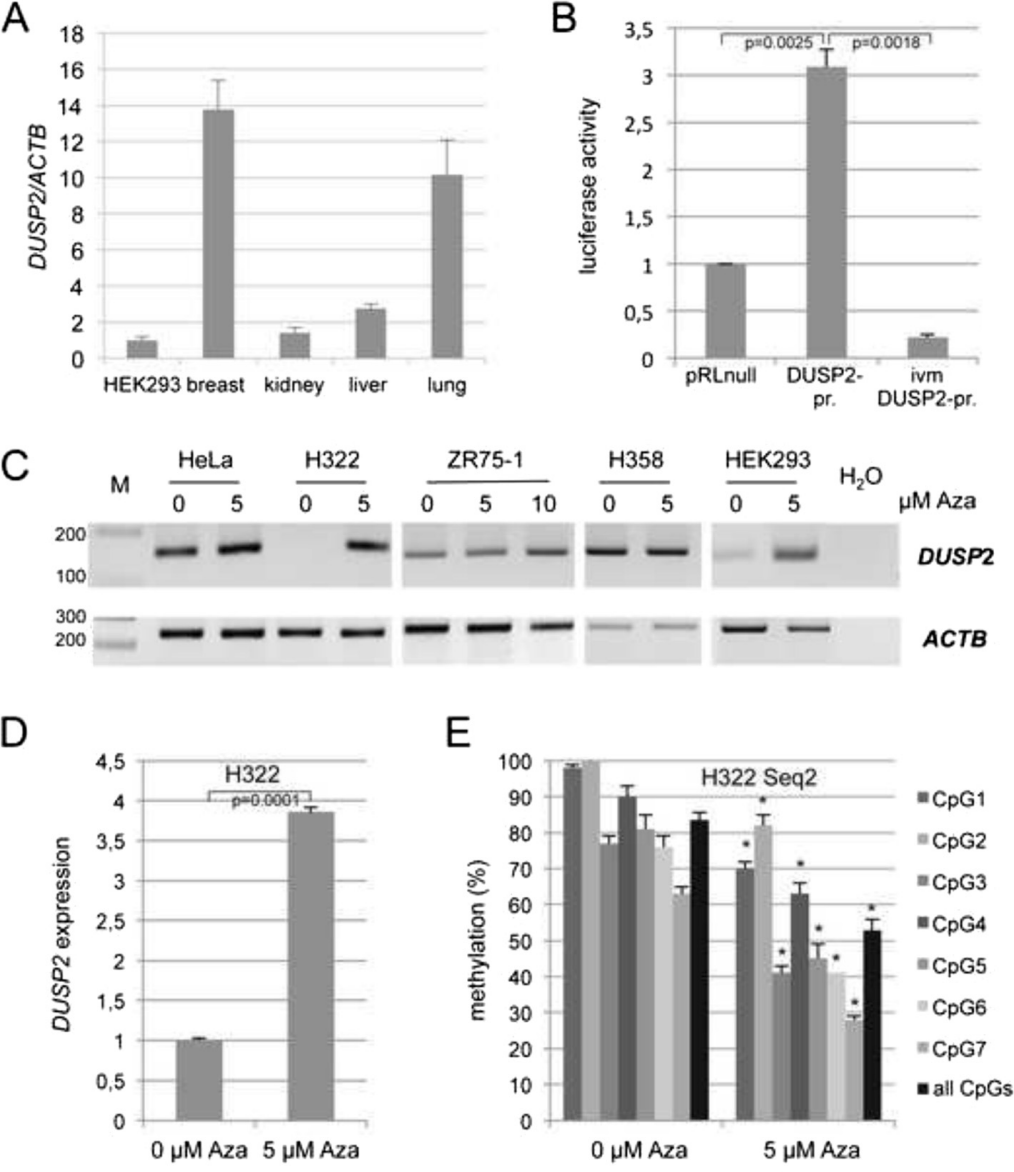
*DUSP2* expression and methylation after 5-Aza-2′-deoxycytidine (Aza) treatment. **a**. Expression of *DUSP2* in normal breast, kidney, liver, lung tissues (Agilent Technologies) and HEK293 cells was analyzed by qRT-PCR and normalized to *ACTB* (HEK293 = 1). **b**. A *DUSP2* promoter fragment (454 bp) was cloned in the pRLnull vector and in vitro methylated (ivm). *DUSP2* promoter constructs (DUSP2-pr.) were transfected in HEK293 cells and expression of *renilla* luciferase was measured and normalized to the expression of the co-transfected *firefly* plasmid pGL3.1 (pRLnull = 1). The analysis included the results (measurement in triplicates) of three independent experiments and significance is indicated (t-test) **c**. Expression analysis of *DUSP2* in several cell lines after treatment with Aza (0, 5 and 10 μM) for four days. Expression of *DUSP2* and *ACTB* was revealed by semi-quantitative RT-PCR and products (136 bp and 226 bp, respectively) were resolved on a 2 % agarose gel with a 100 bp marker ladder (M). **d**. Expression of *DUSP2* in Aza treated H322 cells analyzed by qRT-PCR and normalized to *ACTB*. Data of three independent experiments, whereby each PCR was performed in triplicates and significance is indicated (t-test). **e**. Methylation analysis of *DUSP2* after Aza treatment in H322 cells**.** Methylation of seven CpGs at proximal *DUSP2* transcription start site (Seq2, see also Fig. 1) was analyzed by bisulfite pyrosequencing. Data were calculated from three independent experiments and significance is indicated (* = *p* < 0.01; 0 μM vs. 5 μM Aza)

To further analyze the impact of epigenetic regulation of *DUSP2* in carcinogenesis we investigated its expression in normal tissues and cancer cell lines (Fig. 3). *DUSP2* expression was found in normal breast, kidney, liver, lung tissues and in HEK293 cells (Fig. 3a). In HEK293 cells a genome wide expression array (human ST1.0 S, Affymetrix) detected a 0.02-fold reduced level of *DUSP2* compared to *beta-actin* [41]. We cloned a 454 bp fragment of the *DUSP2* promoter in a luciferase reporter system (Fig. 1a), in vitro methylated (ivm) the construct and analyzed its activity (Fig. 3b). Methylation of the *DUSP2* promoter construct significantly reduced its expression (Fig. 3b). In cancer cells lines (H322, ZR75-1) and HEK293 cells, that exhibit a methylated promoter, expression of *DUSP2* was reduced compared to HeLa and H358 cells, which harbor unmethylated promoter regions (Fig. 3c). H322, ZR75-1 and HEK293 cells were treated with 5-Aza-dC (Aza), a cytidine analogue that inhibits DNA methyltransferases and reactivates epigenetically inactivated TSG [42, 43]. After four days of Aza treatment an induced expression of *DUSP2* was found in H322, ZR75-1 and HEK293 cells (Fig. 3c). However, in HeLa and H358 cells, that harbor unmethylated *DUSP2* promoters, *DUSP2* expression was rather unaffected by Aza (Fig. 3c). For H322 cells the fourfold increased *DUSP2* expression after 5 μM Aza treatment was correlated with a significant 1.6-fold demethylation of seven analyzed CpGs at the *DUSP2* promoter (Fig. 3d and e). Thus, we observed a methylation dependent silencing of *DUSP2* in cancer cell lines, which was reversed by inhibiting DNA methylation.

### Regulation of *DUSP2* by the epigenetic factor CTCF

**Fig. 4:**
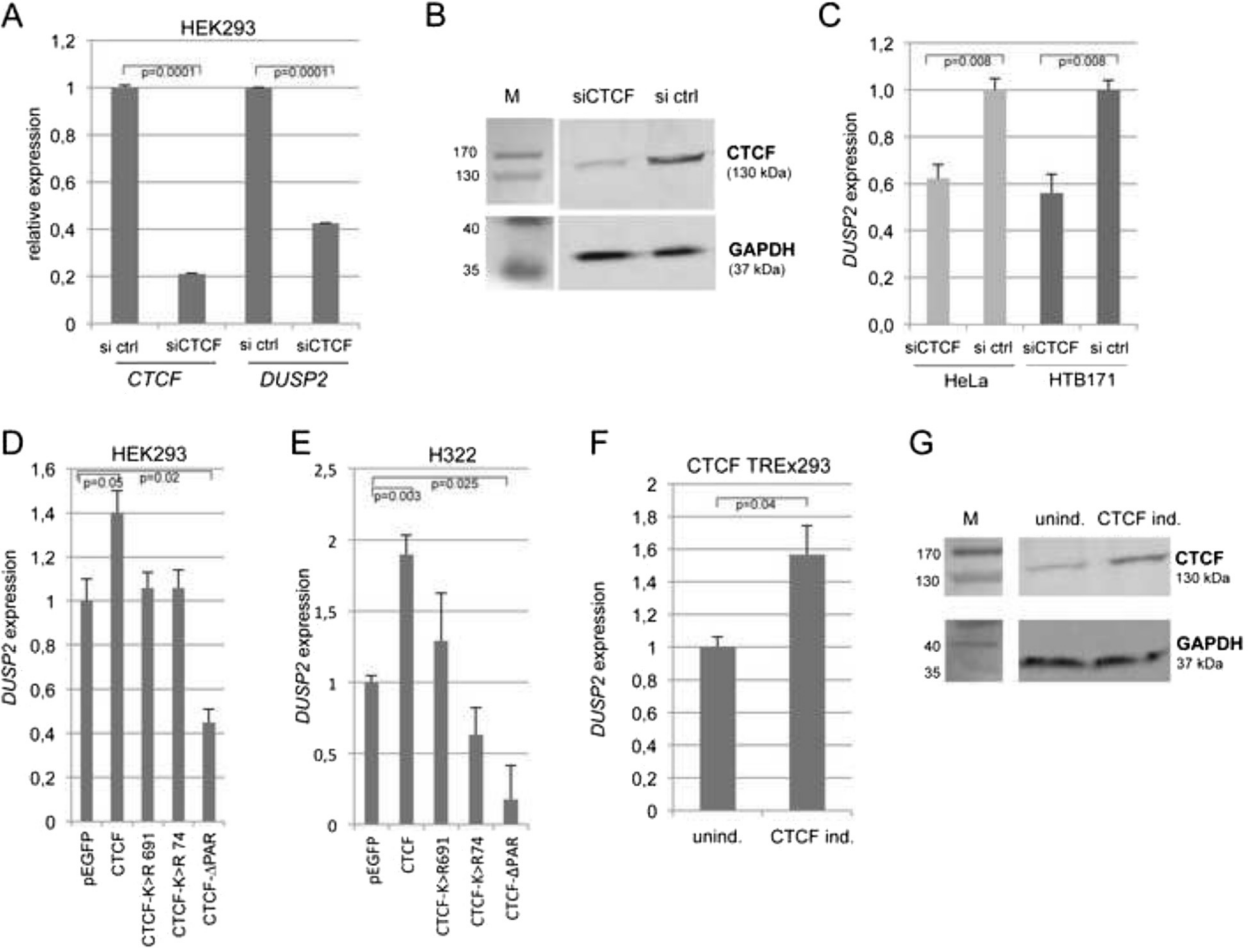
CTCF-dependent expression of *DUSP2.* **a**. HEK293 cells were transfected on two consecutive days with a pool of five different siRNAs against hCTCF (siCTCF) or a control siRNA (si ctrl). After 96 h the RNA was isolated and expression of *CTCF* and *DUSP2* was analyzed by RT-PCR and normalized to *ACTB* (si control = 1). CTCF knockdown was performed 2 times and the qRT-PCR was done in triplicates and significance is indicated (same procedure for C). **b**. Reduction of CTCF protein after RNA interference was analyzed by western blot. GAPDH expression was utilized as control. **c**. Reduction of *DUSP2* expression after siCTCF transfection in HeLa and HTB171 cells. **d**. Different CTCF constructs; wildtype, mutated SUMO site K > R at position 74 (CTCF-K > R74) and 691 (CTCF-K > R691), deletion of CTCF PARylation site (CTCF-ΔPAR) and vector control (pEGFP) were transfected in HEK293. After two days *DUSP2* expression was analyzed by RT-PCR and normalized to *ACTB* (normal control = 1). Triplicates were determined and the data of three independent experiment were averaged and significance was calculated (same procedure for e and f) **e**. Expression of *DUSP2* after transfection of different CTCF constructs in H322 cells (for details see d) **f**. *DUSP2* expression was analyzed in CTCF-inducible TREx293 cells, a stable HEK293 cell line (CTCF TREx293) that allows inducible CTCF expression by tetracycline (5 μg/ml). After two days expression of *DUSP2* was analyzed by RT-PCR and normalized to *ACTB* and compared to the uninduced control (unind. = 1). **g**. Expression of CTCF in TREx293 cells was analyzed by western blot

It has been shown that the insulator binding protein CTCF is involved in the epigenetic regulation of tumor suppressor genes [22–25, 44, 45]. Wendt and Barksi et al*.* have reported that silencing of CTCF by RNA interference caused repression of *DUSP2* in HeLa cells [38, 46]. Database analysis of CTCF ChipSeq Encode data revealed that CTCF binds at the *DUSP2* promoter in HeLa cells (chr2: 96811040-96811190) and a search at the CTCFBSDB2.0 site identified a CTCF motif sequence (GGCAGAGCA chr2: 96811243-96811251) upstream of the *DUSP2* transcription start site (Fig. 1a) [47]. To investigate the role of CTCF in the epigenetic regulation of DUSP2, we performed siRNA mediated knock down of CTCF in HEK293, HeLa and HTB171 cells (Fig. 4). In HEK293, a five-fold reduction of CTCF on RNA and protein levels was accomplished by transfection of CTCF-specific siRNA (Fig. 4a and b). This downregulation of CTCF resulted in a 2.5-fold reduction of *DUSP2* level (Fig. 4a). Knock down of CTCF in HeLa and HTB171 cells induced a 1.7-fold reduction of *DUSP2* (Fig. 4c).

Next, we tested the effect of CTCF overexpression in cancer cells (Fig. 4 and Additional file 4: Figure S2). In HEK293 and H322 cells that harbor a methylated *DUSP2* promoter CTCF transfection resulted in a 1.4- and 2-fold increased expression of *DUSP2*, respectively (Fig. 4d and e). In HeLa cells that exhibit an unmethylated promoter this CTCF-induced expression of *DUSP2* was absent (Additional file 4: Figure S2A). The testis-specific paralogue of CTCF, termed CTCFL or BORIS was unable to induce *DUSP2* expression in HEK293 cells (Additional file 4: Figure S2B). Previously, it has been reported that SUMOylation and PARylation of CTCF are involved in its regulatory function [23, 48, 49]. Therefore we generated different CTCF constructs that lack either the N- or C-terminal SUMOylation site (K > R substitution) at position 74 (CTCF-K > R74) and 691 (CTCF-K > R691), respectively or harbor a deletion of its PARylation sites from position 216 to 243 (CTCF-ΔPAR). Expression of the two SUMOylation-site deficient CTCF constructs in HEK293 and H322 cells resulted in a lack of *DUSP2* induction compared to wildtype CTCF (Fig. 4d and e). Transfection of the PARylation site deficient CTCF construct (CTCF-ΔPAR) downregulated *DUSP2* expression significantly (2.2- and 5.6-fold, respectively) compared to the vector control in HEK293 and H322 cells (Fig. 4d and e). Furthermore, we have generated a stable HEK293 cell line (CTCF TREx293) that allows tetracycline-inducible CTCF expression (Fig. 4f and g). Induction of CTCF in TREx293 cells resulted in 1.6-fold higher *DUSP2* expression (Fig. 4f).

### Increased CTCF binding at the *DUSP2* promoter is associated with reduction in methylation levels and induced *DUSP2* expression

**Fig. 5:**
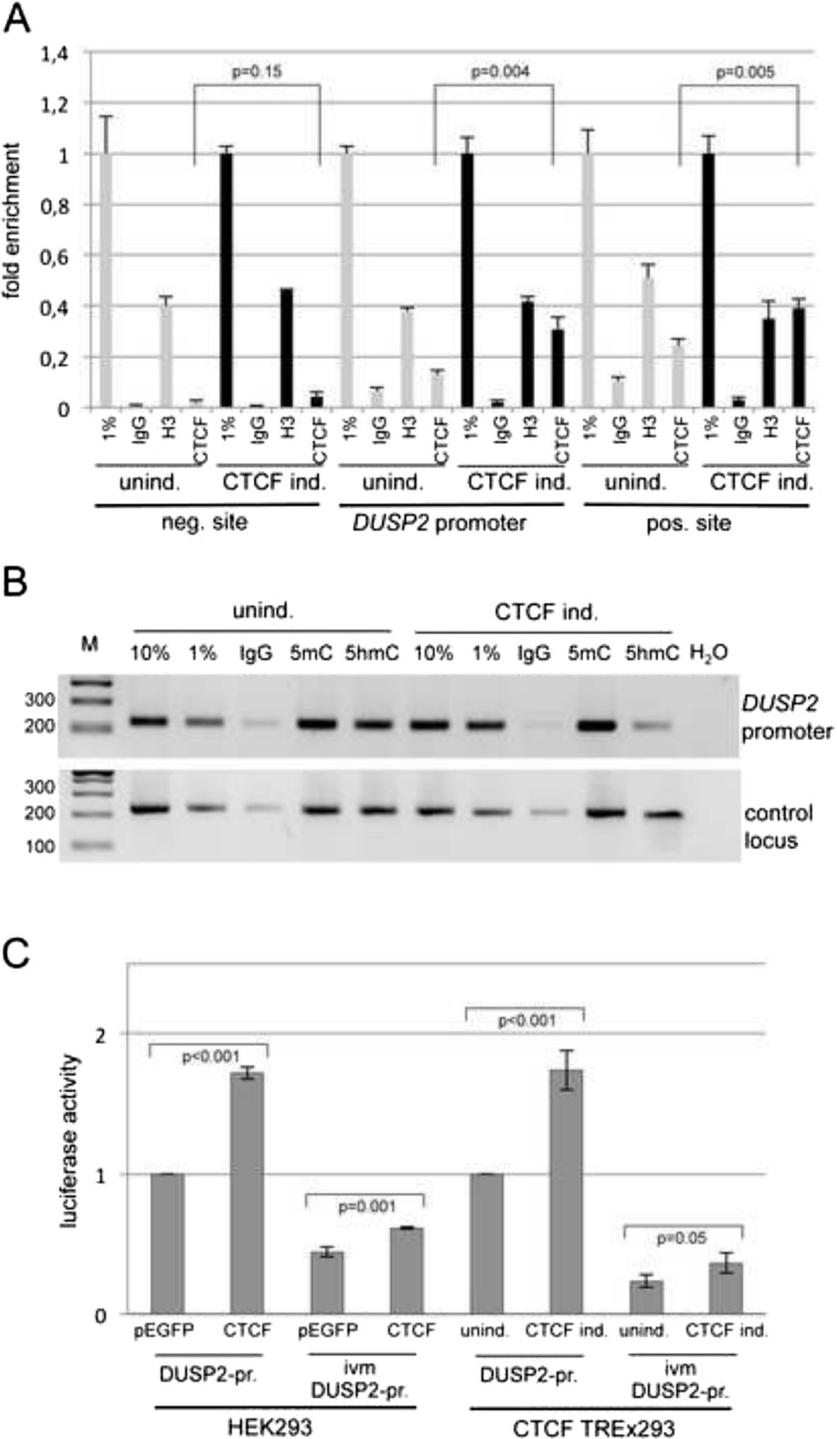
Epigenetic regulation of the *DUSP2* promoter by CTCF. **a**. Binding of CTCF at the *DUSP2* promoter analyzed by quantitative ChIP. CTCF expression was induced in CTCF TREx293 cells by tetracycline (5 μg/ml) for 48 h and uninduced cells were used as control. The chromatin was prepared, precipitated with a CTCF-, histone H3- or control IgG-antibodies and amplified with gene specific primers for the *DUSP2* promoter, a negative site and a *bona fide* positive site within the *DUSP2* locus. CTCF binding was quantified by qPCR (triplicates from 2 independent experiments) and significance was calculated. Values of the precipitated sample were normalized to 1 % input (=1). **b**. Methyl-DNA immunoprecipitation (MeDIP) analysis of the *DUSP2* promoter. MeDIP with 5mC- and 5hmC-antibodies was done with DNA from uninduced or induced CTCF TREx293 cells. The detection of the 5hmC and 5mC level was performed with semi-quantitative PCR of the *DUSP2* promoter and a control locus. PCR products were separated together with a 100 bp marker (M) in 2 % agarose gel. **c**. Effect of CTCF on the *DUSP2* promoter. A *DUSP2* promoter fragment (454 bp) was cloned in the pRLnull vector and in vitro methylated (ivm). 2.7 μg *DUSP2* promoter constructs (DUSP2-pr.) were transfected in HEK293 or CTCF TREx293 cells and GFP-CTCF or GFP vector (1 μg each) was co-transfected or CTCF was induced for 24 h, respectively. Expression of *renilla* luciferase was measured and normalized to the expression control vector or to the co-transfected *firefly* plasmid pGL3.1 (300 ng), respectively. Significance is indicated (t-test)

To analyze the mechanism of CTCF regulated *DUSP2* expression in detail, we analyzed the binding of CTCF at the *DUSP2* locus in CTCF TREx293 cells by ChIP (Fig. 5a). Therefore, we utilized CTCF and histone H3 antibody and quantified the precipitation of the *DUSP2* promoter, a *bona fide* CTCF target site at 1 kb downstream (positive site) and negative site 1 kb upstream of the DUSP2 transcriptional start site by qPCR. After CTCF induction we observed a significant 2.3-fold increased binding of CTCF at the *DUSP2* promoter. Histone H3 binding and CTCF binding at the negative site were not altered. At the positive site binding of CTCF was significantly increased by 1.6 times after tetracycline-induced CTCF expression (Fig. 5a). Histone H3 levels were reduced after CTCF induction at the positive site (Fig. 5a).

Subsequently, we analyzed changes in DNA methylation after CTCF induction at the DUSP2 promoter (Fig. 5). Conventional bisulfite sequencing is not able to distinguish between 5-methylcytosine (5mC) or 5-hydroxy-methylcytosine (5hmC) levels. Therefore we precipitated different DNA regions with antibodies that bind 5mC or 5hmC in induced or uninduced CTCF TREx293 cells. Interestingly, after CTCF induction we observed a decrease in 5hmC-precipitated *DUSP2* promoter sequences compared to a control locus control (chr5q14.1) (Fig. 5b). This result suggests that CTCF induces dehydroxy-methylation of the *DUSP2* promoter. To analyze the effect of CTCF on the *DUSP2* promoter, we performed luciferase reporter assays (Fig. 5c). Therefore the *DUSP2* promoter construct was in vitro methylated (ivm) and transfected together with CTCF in HEK293 cells. CTCF transfection significantly induced luciferase reporter activity of the unmethylated *DUSP*2 promoter (1.7-fold) and ivm *DUSP2* promoter (1.4-fold) compared to the pEGFP control vector (Fig. 5c). Moreover CTCF induction in CTCF TREx293 cells resulted in a 1.7-fold (unmethylated) or 1.5-fold (ivm) increased *DUSP2* promoter activity compared to uninduced cells (Fig. 5c). Taken together these data suggest that CTCF epigenetically activates *DUSP2* expression.

## Discussion

Previously it has been reported that *DUSP2* expression is downregulated in many human cancers [8]. However the mechanism of its silencing was not analyzed in details. Deletion of the *DUSP2* locus at 2q11.2 is rather infrequent in cancer. Here we show that the promoter of *DUSP2* is hypermethylated in different human cancer cell lines including lung, breast and skin cancers and in HEK293 cells (Figs. 1 and 2). In primary Merkel cell cancer (MCC) we observed a significant tumor specific methylation of *DUSP2*. MCC is one of the most aggressive cancers of the skin and we have reported frequent hypermethylation of the Ras Association Family Members RASSF1A and RASSF10 in this tumor entity [28, 29]. Hypermethylation of DUSP2 and murine Dusp2 and has been reported in breast cancer cell lines, however methylation in primary human mammary tumors was absent [50], which was also observed in our study (Additional file 3: Figure S1). By inhibiting DNA methyltransferases with the cytidine analogue 5-aza-dC we found that the *DUSP2* gene is epigenetically reactivated by its demethylation (Fig. 3). The promoter methylation of *DUSP2* in HEK293 consists of 5mC and 5hmC epigenetic marks (Fig. 5). Additionally, we have revealed that CTCF reactivates *DUSP2* and this is associated with demethylation of its CpG island promoter (Figs. 4 and 5b). CTCF is a DNA binding factor well known for its multiple functions in gene regulation. Depending on the participating genetic locus it is involved in transcriptional activation [51–53], transcriptional repression [54, 55] or enhancer blocking [56]. Here we show increased binding of CTCF to the *DUSP2* promoter (Fig. 5a) and CTCF-dependent induction of the *DUSP2* promoter activity (Fig. 5c). Thus it will be interesting to analyze the exact mechanism of CTCF induced *DUSP2* expression and promoter dehydroyx-methylation in further details.

DUSP2 encodes a dual-specificity phosphatase that inactivates ERK1/2 and p38 MAPK [4, 5]. DUSP2 has also been found to regulate p53- and E2F1-regulated apoptosis [6, 7]. Dual-specificity phosphatases are negative regulators of the MAPK signal transduction, proliferative pathways that are often activated in cancers [1]. Downregulation of *DUSP2* was detected in human acute leukemia coupled with activation of MEK and hyperexpression of ERK [9]. Especially in acute myeloid leukemia, translocation and mutations of TET1 and TET2 gene are frequently observed [57–59]. Moreover it has been reported that CTCF binds TET proteins [21]. Thus it will be interesting to analyze if TET proteins are directly involved in the epigenetic regulation of *DUSP2*. Here we observed increased binding of CTCF to its target site in the *DUSP2* promoter after CTCF induction (Fig. 5a). This binding may alter distinct TET- or DNMT-associated chromatin complexes at the *DUSP2* promoter region involved in CTCF-regulated DNA methylation as previously reported [21, 25, 60, 61] and revealed in Fig. 5b. However CTCF-dependent regulation of *DUSP2* may also involve its function in chromosome configuration, chromatin insulation or transcriptional regulation [62, 63].

We also observed that the CTCF paralogue CTCFL/BORIS was unable to reactivate *DUSP2* expression (Additional file 4: Figure S2). Since the cancer-testis specific BORIS is aberrantly expressed in cancer, an oncogenic role for BORIS has been proposed [64, 65]. It was reported that CTCF, unlike BORIS, cannot bind to methylated binding sites [66]. Therefore, it is interesting to note that the CTCF consensus site in the *DUSP2* promoter sequence lacks CpG sites (Fig. 1a) and ChIP data show an enhanced CTCF binding in CTCF induced TREx293 cells at this site (Fig. 5a). It was also postulated that CTCF itself acts as a tumor suppressor [26, 27]. CTCF contains a N-terminal PARylation site [49]. Here we observed that overexpression of CTCF lacking its PARylation site resulted in repression of *DUSP2* expression in H322 and HEK293 cells (Fig. 4d and e). This result suggests that the PARylation site of CTCF is important for its activating function. It has been reported that defective CTCF PARylation and dissociation from the molecular chaperone nucleolin occurs in *CDKN2A*- and *CDH1*-silenced cells, abrogating its TSG function [23]. Using CTCF mutants, the requirement of PARylation for optimal CTCF function in transcriptional activation of the p19ARF promoter and inhibition of cell proliferation has been demonstrated [49]. In this model CTCF and Poly(ADP-ribose) polymerase 1 form functional complexes [49]. Furthermore, CTCF contains two SUMOylation sites [48]. Overexpresssion of the CTCF construct with mutated SUMOylation sites in the CTCF N-terminus or C-terminus resulted in a lack of *DUSP2* reactivation in the lung cancer H322 and HEK293 cells (Fig. 4d and e). SUMOylation of CTCF has been associated with its tumor suppressive function in *c-myc* expression [48]. There is also a report that CTCF SUMOylation modulates a CTCF domain, which activates transcription and decondenses chromatin [67].

## Conclusions

Downregulation of the negative regulator *DUSP2* of the oncogenic MAPK signaling pathway has been reported in cancer. In the present study we have investigated the epigenetic regulation of *DUSP2* in detail and we show that *DUSP2* is epigenetically silenced by promoter methylation in human cancer. Especially in primary Merkel cell carcinoma a tumor-specific hypermethylation of *DUSP2* was revealed. Thus it will be interesting to further analyzed primary tumor tissues regarding an aberrant *DUSP2* promoter hypermethylation. Moreover our data indicate that the insulator-binding factor CTCF is involved in the epigenetic regulation of *DUSP2*. Further research will elucidate the exact mechanism of the CTCF-mediated induction of *DUSP2*.

## Additional files

Additional file 1: Table S1. List of primers for RT-PCR. (DOCX 17 kb) Additional file 2: Table S2. Primers for site directed mutagenesis of CTCF. (DOCX 47 kb) Additional file 3: Figure S1. Four examples of methylation analysis of *DUSP2* in primary pheochromocytomas (Pheo), small cell lung cancer (SCLC) and breast cancer (BrCa). (PDF 2955 kb) Additional file 4: Figure S2. CTCF- and BORIS‐dependent expression of *DUSP2* in HeLa and HEK293 cells, respectively *A*. (PDF 1891 kb)

## Abbreviations

Aza: 5-aza-dC
COBRA: combined bisulfite restriction analysis
CTCF: CCCTC binding factor
DUSP2: dual specificity phosphatase 2
MCC: Merkel cell carcinoma
