# Supplementary material for: The dual specificity phosphatase 2 gene is hypermethylated in human cancer and regulated by epigenetic mechanisms
Source: BMC Cancer. 2016 Feb 1;16:49. doi: 10.1186/s12885-016-2087-6 (PMC4736155; doi:10.1186/s12885-016-2087-6)
Supplement: Additional file 1: Table S1. — List of primers for RT-PCR. (DOCX 17 kb) [file 12885_2016_2087_MOESM1_ESM.docx]

| Primer | **Sequence (5’-3’)** | **Use** |
| --- | --- | --- |
| DUSP2RTF1 | GCCAGCTGCCCCAACCACTTTG | DUSP2 |
| DUSP2RTR1 | GGCCTCCGCTGTTCTTCACCCA | DUSP2 |
| ßACTF | CCTTCCTTCCTGGGCATGGAGTC | ACTB |
| ßACTR | CGGAGTACTTGCGCTCAGGAGGA | ACTB |
| GGCTCFRTFW | CAGGAAACGGAGGCTACGGTGG | CTCF |
| GGCTCFRTRV | CCTCCTGCAGGCCTCCTTTGGA | CTCF |
| hCTCFFW | TTACACGTGTCCACGGCGTTC | FL CTCF |
| hCTCFRV | GCTTGTATGTGTCCCTGCTGGCA | FL CTCF |
| CTCFCTRTF1 | TGGCCGAAAGAGGAAGATGCGC | CT CTCF |
| CTCFCTRTR1 | GGTGGTGCAGGAGCCTCTGGCT | CT CTCF |
| CTCFZFRTF1 | CGCCGTTCCAACCTGGACCG | ZF CTCF |
| CTCFZFRTR1 | TCTGGGCACTTGTGAGGGCGAG | ZF CTCF |
| BORISRTFW | GGCAAAGGCTTTTCCCGCTGG | BORIS |
| BORISRTRV | TTCGCGGCTTCCTT CCATCCC | BORIS |

**Supplement Table 1.** List of primers for RT-PCR
