# Supplementary material for: The dual specificity phosphatase 2 gene is hypermethylated in human cancer and regulated by epigenetic mechanisms
Source: BMC Cancer. 2016 Feb 1;16:49. doi: 10.1186/s12885-016-2087-6 (PMC4736155; doi:10.1186/s12885-016-2087-6)
Supplement: Additional file 2: Table S2. — Primers for site directed mutagenesis of CTCF. (DOCX 47 kb) [file 12885_2016_2087_MOESM2_ESM.docx]

| CTCFMUTK>RS1 | Ccaactctccttcaaatgaggactgaagtaatggaaggt | K>R substitution at position 74 |
| --- | --- | --- |
| CTCFMUTK>RA1 | Accttccattacttcagtcctcatttgaaggagagttgg |  |
| CTCFSUMO2MutU | CgaaaacattatagtagaagtaaggaaagaacctgatgcagaaacaG | K>R substitution at position 691 |
| CTCFSUMO2MutL | Ctgtttctgcatcaggttctttccttacttctactataatgttttcg |  |
| CTCFMUTDPARS2 | AAAGAGTAAGCTTCGCTACACCAAGGTGGTGGGCAACATGAAAC | Deletion of the PARylation site at position 216 to 243 |
| CTCFMUTDPARA2 | GTTTCATGTTGCCCACCACCTTGGTGTAGCGAAGCTTACTCTTT |  |

**Supplementary Table S2**. Primers for site directed mutagenesis of CTCF
