# Supplementary material for: The dual specificity phosphatase 2 gene is hypermethylated in human cancer and regulated by epigenetic mechanisms
Source: BMC Cancer. 2016 Feb 1;16:49. doi: 10.1186/s12885-016-2087-6 (PMC4736155; doi:10.1186/s12885-016-2087-6)
Supplement: Additional file 3: Figure S1. — Four examples of methylation analysis of DUSP2 in primary pheochromocytomas (Pheo), small cell lung cancer (SCLC) and breast cancer (BrCa). (PDF 2955 kb) [file 12885_2016_2087_MOESM3_ESM.pdf]

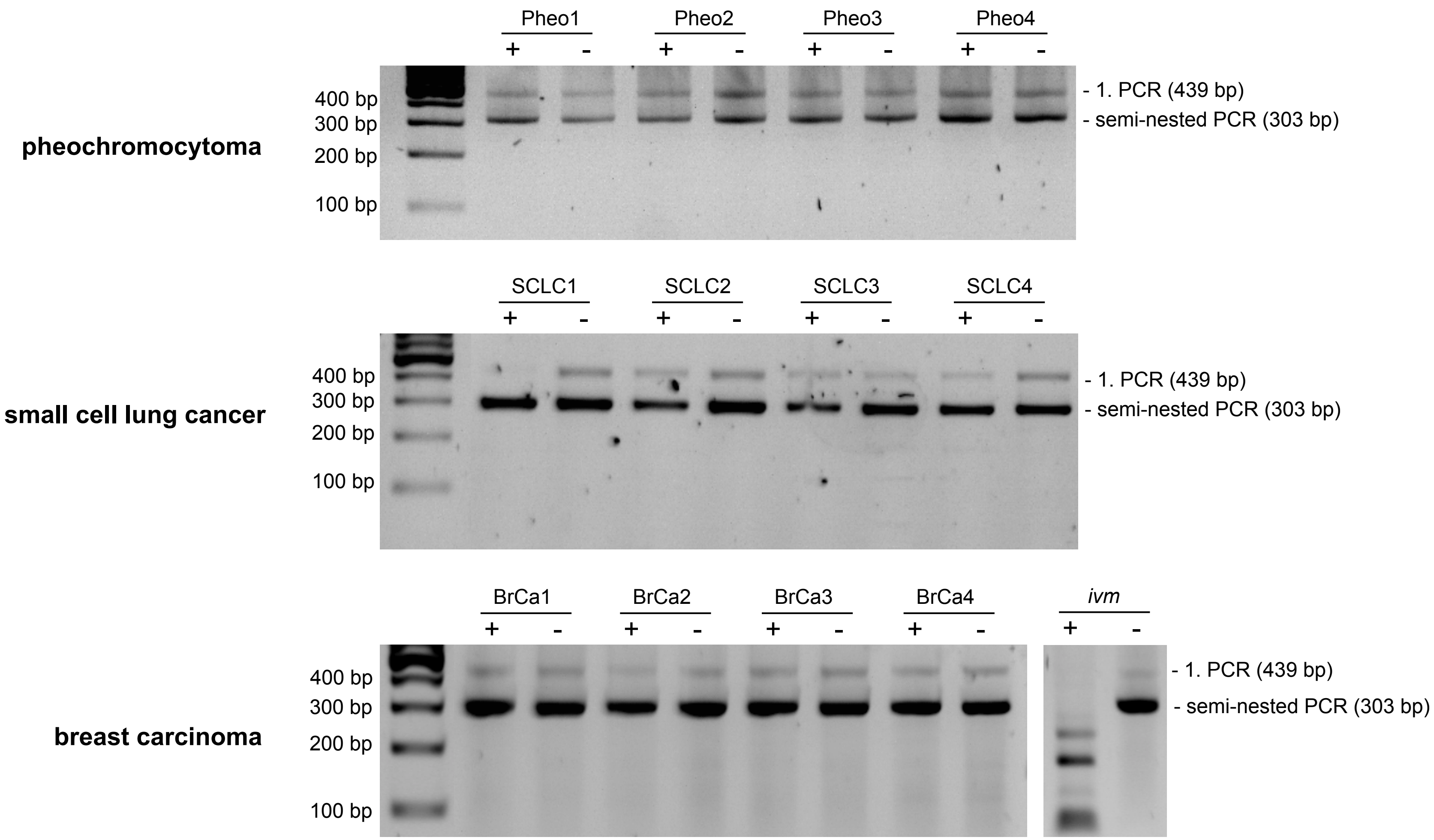

Supplement Figure S1. Four examples of methylation analysis of *DUSP2* in primary pheochromocytomas (Pheo), small cell lung cancer (SCLC) and breast cancer (BrCa). For COBRA bisulfite-treated DNA from four primary cancers and *in vitro* methylated HeLa DNA (*ivm*) was amplified by semi-nested PCR. First and second PCR products are indicated (439 bp and 303 bp, respectively). Products were digested with *Taq*I (+) or mock digested (-) and resolved on 2% agarose gels with a 100 bp marker (M).
