# Supplementary material for: The dual specificity phosphatase 2 gene is hypermethylated in human cancer and regulated by epigenetic mechanisms
Source: BMC Cancer. 2016 Feb 1;16:49. doi: 10.1186/s12885-016-2087-6 (PMC4736155; doi:10.1186/s12885-016-2087-6)
Supplement: Additional file 4: Figure S2. — CTCF- and BORIS‐dependent expression of DUSP2 in HeLa and HEK293 cells, respectively A. (PDF 1891 kb) [file 12885_2016_2087_MOESM4_ESM.pdf]

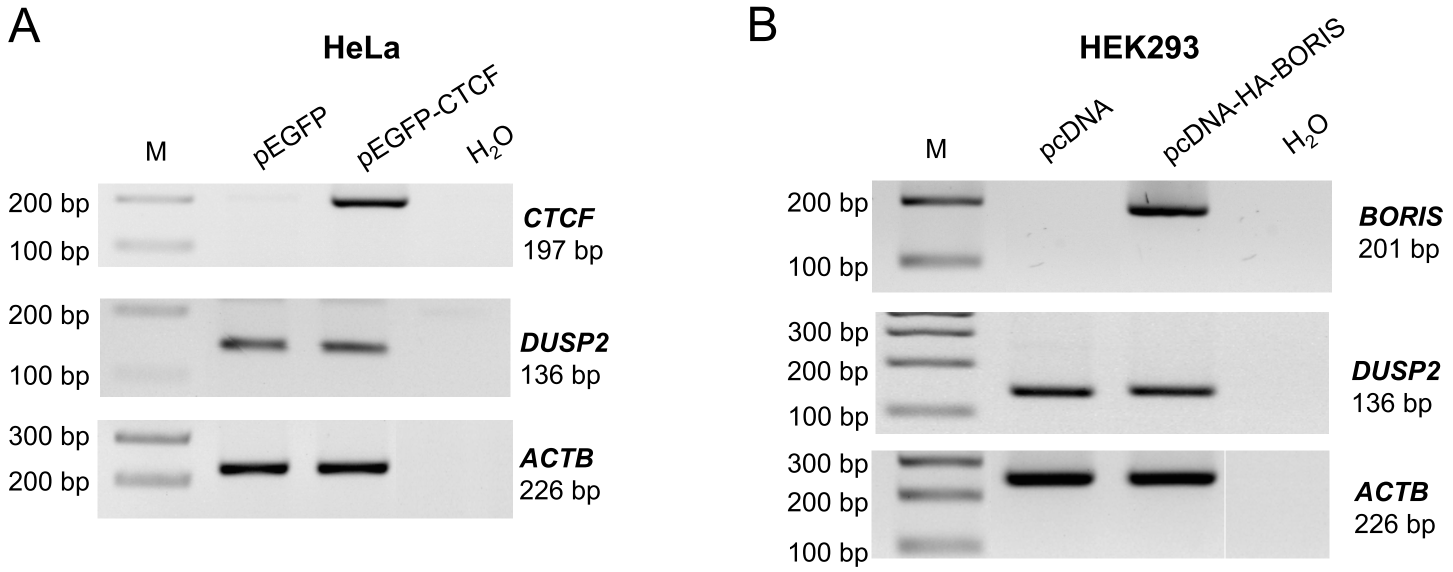

Supplement Figure S2. CTCF- and BORIS-dependent expression of *DUSP2* in HeLa and HEK293 cells, respectively. *A*. CTCF and vector control (pEGFP) were transfected in HeLa. After two days *CTCF, DUSP2* and *ACTB* expression was analyzed by RT-PCR and separated on a 2% gel together with a 100 bp ladder marker (M) **B.** Expression of *DUSP2* after transfection of BORIS and vector control in HEK293 cells (for details see A).
